# Variation of QRS morphology of premature ventricular contractions originate from the left‐ventricular outflow tract during ablation

**DOI:** 10.1111/jce.14228

**Published:** 2019-10-22

**Authors:** Liu Qifang, Tian Ye, Jiang Zhi, Huang Jing, Zhao Yidong, Yang Long

**Affiliations:** ^1^ Department of Cardiology Guizhou Provincial People's Hospital Guiyang China

**Keywords:** conduction impairment, left‐ventricular outflow tract, local potentials, premature ventricular contractions, radiofrequency ablation

## Abstract

Idiopathic ventricular arrhythmias originating from the aortic sinus of Valsalva often show preferential conduction to the right‐ventricular outflow tract, which may render radiofrequency ablation more difficult. We describe a patient with symptomatic premature ventricular contractions of left‐ventricular outflow tract origin presenting with a variation of QRS morphology during ablation. The correlation between the characteristics of local voltage potentials and the real origin site of the ventricular arrhythmia is discussed.

## 1 CASE REPORT

**Figure 1:**
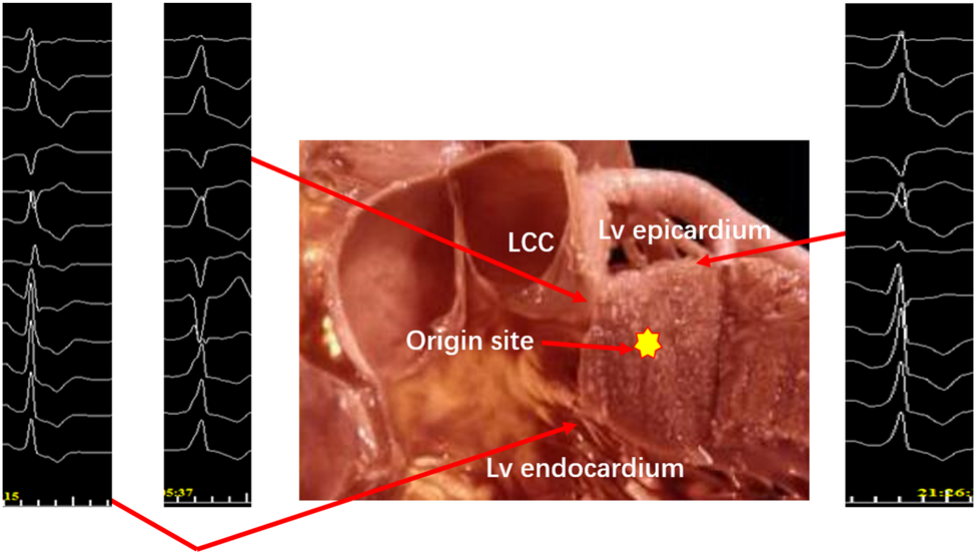
Twelve‐lead electrocardiograms of PVCs and a long axis section of the LV showing the presumed ventricular arrhythmia origin (yellow star) and breakout sites (red arrows). LCC, left coronary cusp; LV, left ventricular; PVC, premature ventricular contraction [Correction added on 21 November 2019, after first online publication: Figure 1 has been replaced with a new version of the figure]

A 45‐year‐old woman visited our hospital for a palpitation that arose owing to frequent premature ventricular contractions (PVCs). The QRS morphology of the PVCs was characterized by an r pattern with a notch in lead I, an rS pattern in leads V1 and V2, a QS pattern in leads aVR and aVL, and a precordial transition in lead V3 (Figure 1). On 24‐hour Holter monitor recordings, the PVC burden was 27 834. We decided to perform catheter ablation, as antiarrhythmic medications were not useful for managing this patient's symptomatic PVCs.

**Figure 2:**
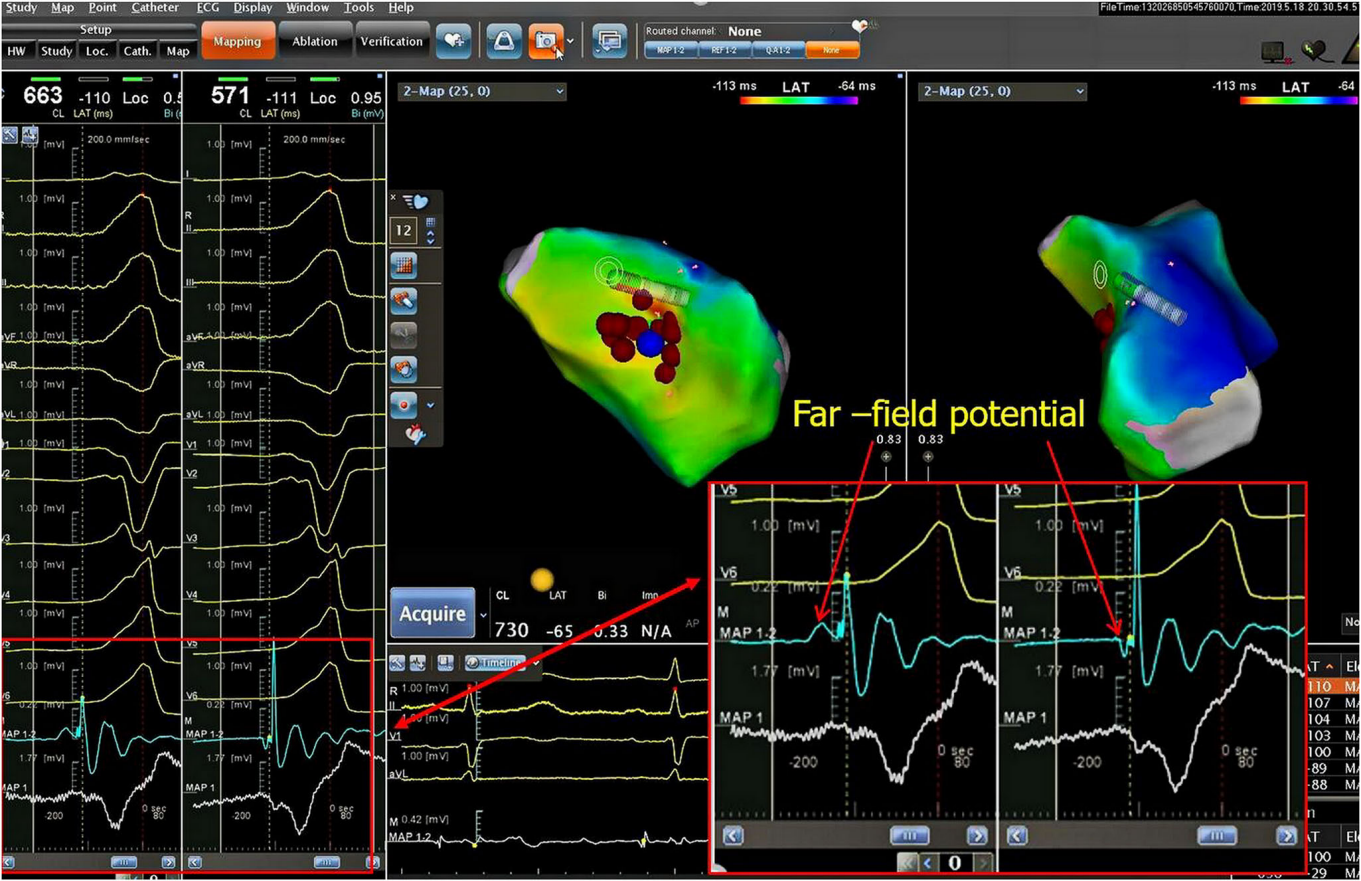
The ablation site of RVOT. The earliest onset of the bipolar electrogram showed a slow‐frequency and low‐amplitude far‐field potential in RVOT (red arrows) and the unipolar electrogram showed an rS pattern. The local ventricular activation was timed at the maximal negative *dV/dt* of the unipolar electrogram and the maximal amplitude of the bipolar electrogram. RVOT, right‐ventricular outflow tract

**Figure 3:**
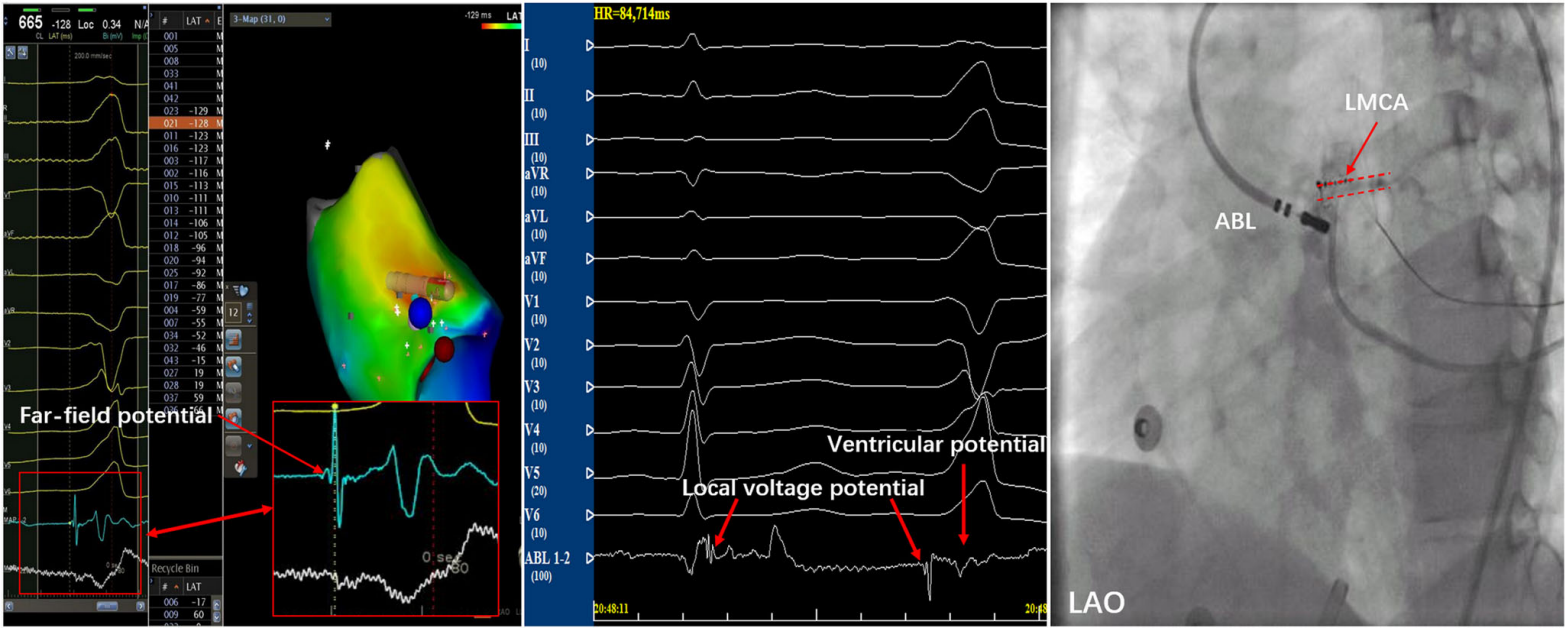
Intracardiac electrograms demonstrating the LVP. The bipolar electrogram exhibited the LVP (red arrows) following the ventricular potential during sinus rhythm and had an inversion of the sequence and polarity during the PVC. Fluoroscopic images showed that the ABL was close to the ostium of the LMCA. ABL, ablation catheter; LAO, left anterior oblique; LMCA, left main coronary artery; LVP, local voltage potential

**Figure 4:**
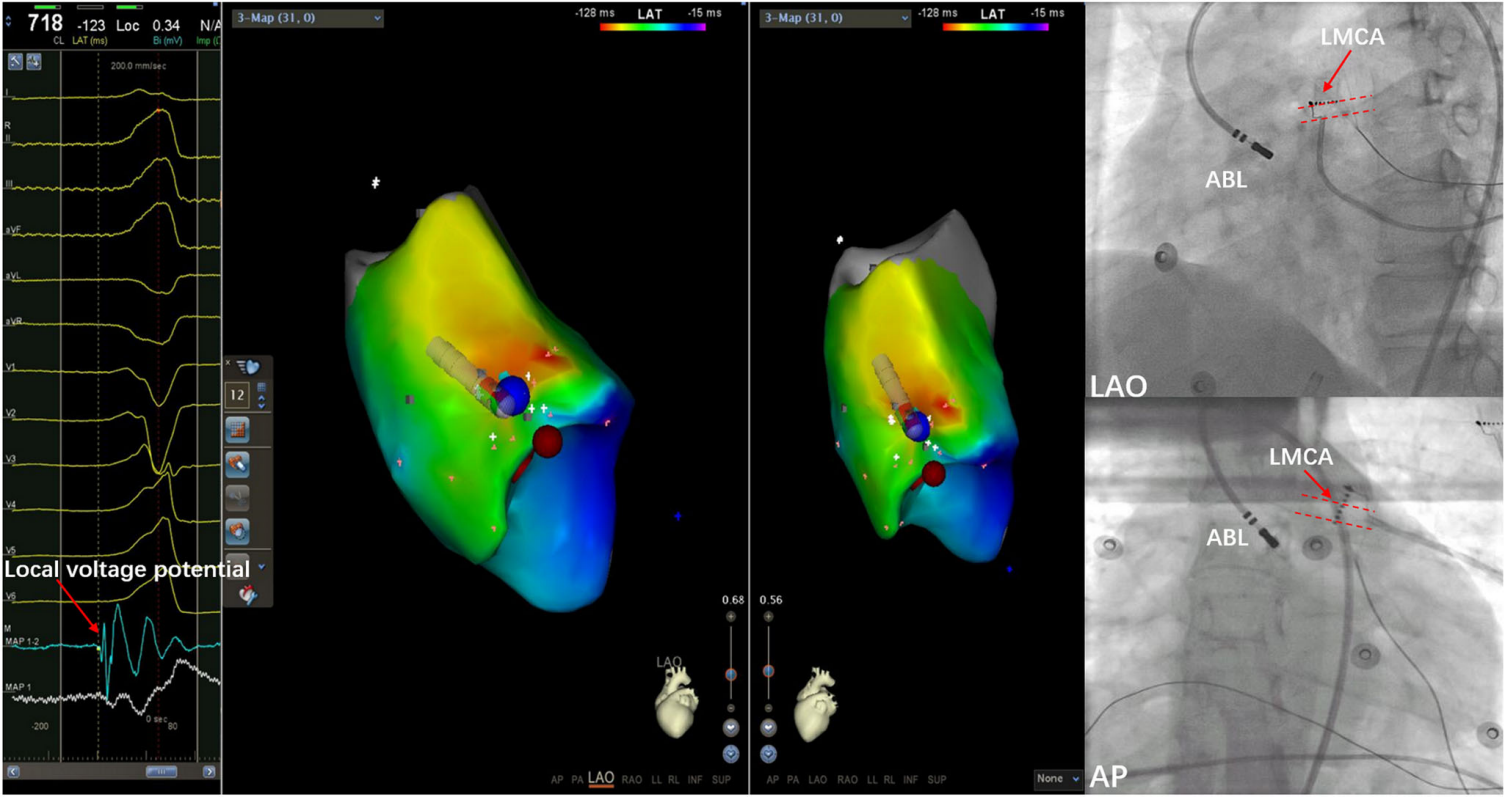
The ablation site of the left sinus of Valsalva. The bipolar electrogram showed no isoelectric line between LVP and the ventricular potential and the unipolar electrogram showed a QS pattern. Fluoroscopic images showed that the ABL was far away from the ostium of the LMCA. ABL, ablation catheter; AP, anteroposterior position; LAO, left anterior oblique; LMCA, left main coronary artery; LVP, local voltage potential

**Figure 5:**
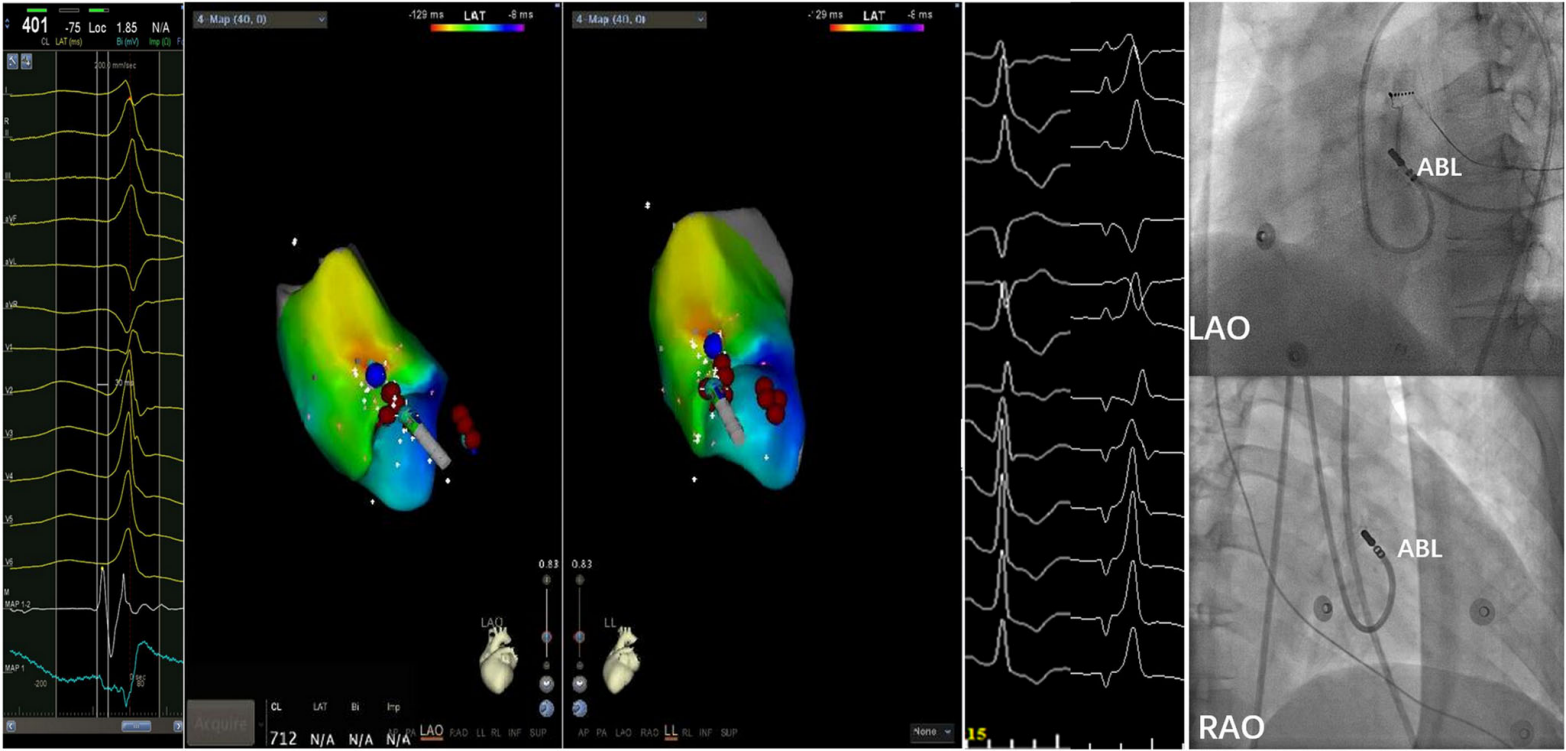
The ablation site of the subaortic valvular region. The local ventricular activation preceded the onset of the QRS by 30 milliseconds and pace mapping showed an excellent match of the QRS morphology at the ablation site. ABL, ablation catheter; LAO, left anterior oblique; RAO, right anterior oblique

**Figure 6:**
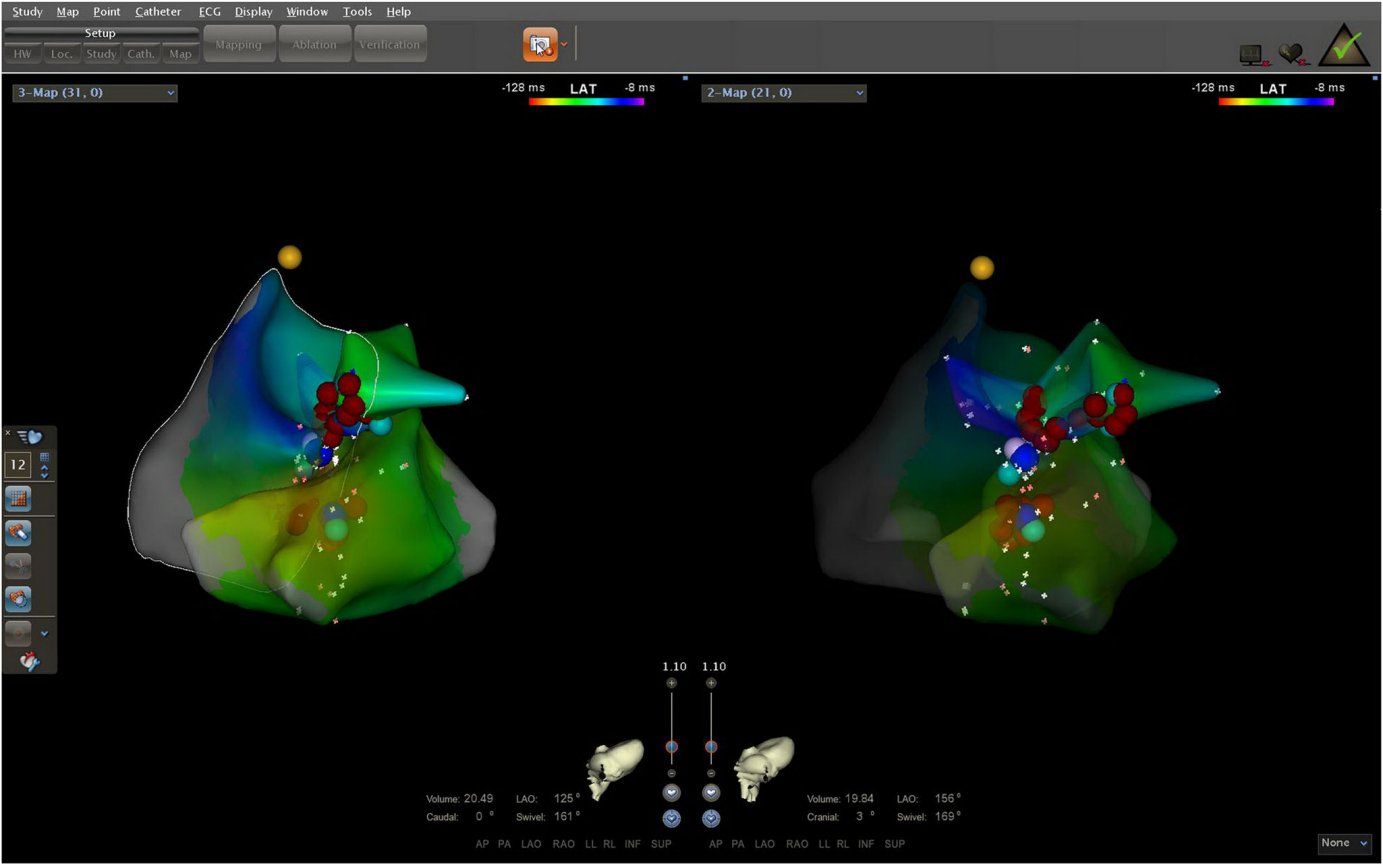
Ablation site including right‐ventricular outflow tract, the left sinus of Valsalva, and the subaortic valvular region

Mapping and ablation were performed using an ablation catheter introduced through a long sheath via the left femoral artery and right femoral vein. Local activation was determined based on a combination of the unipolar and bipolar electrograms and timed at the maximal negative *dV/dt* of the local unipolar electrogram. First, the electrocardiogram (ECG) features suggested that the PVCs originated from the right‐ventricular outflow tract (RVOT). Activation mapping was performed in the RVOT during the PVCs. At the earliest ventricular activation (EVA) site in RVOT, the local ventricular potential was recorded for 42 milliseconds before the earliest start of the QRS complexes during clinical PVCs; this was preceded by a slow, low‐amplitude, far‐field potential which was recorded for 53 milliseconds before the earliest start of the QRS (Figure 2). This phenomenon suggested that the origin of the arrhythmia was not in the RVOT. Rather, the arrhythmia should be in the left‐ventricular outflow tract (LVOT). Radiofrequency (RF) with a target temperature of 43°C and a maximum power output of 40 W was delivered at the EVA site in the RVOT. PVCs could not be successfully eliminated by two RF applications. Activation mapping was added in the aortic sinus of Valsalva during the PVCs. We found that the electrical impulse of the sinus beat bringing the discrete local voltage potential (LVP) was recorded at the end of the QRS complex. When the PVC fired, we found that the inversion of the LVP and polarity of the LVP became visible. Interestingly, there was an isoelectric line between the LVP and the ventricular potential, and the conduction velocity was so slow that the EVA site was recorded for 59 milliseconds before the earliest start of the QRS complexes in the aortic sinus of Valsalva. RF catheter ablation (RFCA) was not applied at the EVA site of the aortic sinus of Valsalva for the onset of the bipolar electrogram was far‐field potential and the mapping site was close to the ostium of the left main coronary artery. RF energy application at this site could not eliminate the PVCs and possibly resulted in a lesion to artery. (Figure 3) (Video S1). Far away from the ostium of the left main coronary artery, we detected an LVP preceding the earliest start of the QRS complexes for 56 milliseconds and no isoelectric line between the LVP and the ventricular potential in the PVCs (Figure 4). The RFCA (maximum power of 40 W, temperature cut‐off of 42°C) was delivered in this area. Of note, changes in the QRS morphology of the PVCs occurred after ablation. The r wave in leads I changed to an RS wave and the QS wave in lead V1 changed to an R wave. Moreover, the precordial R‐wave transition shifted from lead V3 to V1 after ablation, suggesting alterations in the breakout site from the left sinus of Valsalva to the subaortic valvular region (Figure 1). Activation mapping was performed in the subaortic valvular region and a far‐field ventricular prepotential that had preceded QRS onset was recorded. Pace mapping was performed at a pacing cycle length of 500 milliseconds at the subaortic valvular region; the pace map score in this site was very good (Figure 5). Ablation was applied in the subaortic valvular region and the PVCs with the second QRS morphology were suppressed; however, the ablation could not terminate the spontaneous PVCs. Furthermore, the QRS morphology of the PVCs changed to the third form (Video S2). This showed a wider pseudo delta wave, a higher maximum deflection index, and a longer intrinsicoid deflection time. Mapping of the great cardiac vein (GCV) should have been added, but the catheter could not be delivered in GCV due to an anatomic distortion. The ablation was delivered sequentially at the left sinus of Valsalva, RVOT, and subaortic valve irrespective of activation time (Figure 6). After ablation, the patient received isoproterenol infusion and was monitored for 30 minutes. The PVCs did not recur. In 3 months, Holter ECG monitoring revealed no ventricular arrhythmias.

## 2 DISCUSSION

There have been a few reports describing ventricular tachycardias (VTs) of the aortic sinus of Valsalva origin and breakout sites in the RVOT.^1^ Yamada et al reported how PVCs originating from the LVOT exhibit variable ECG during mapping and ablation, and suggested that preferential conduction to multiple exits may occur in the LVOT.^2^ In this case, when the RFCA was sequentially applied in the left sinus of Valsalva and the subaortic valvular region, the QRS morphology of the PVCs changed from one type of ECG characteristic to another. These findings suggest an intramural origin with preferential conduction to multiple exit sites and were consistent with the findings of previous reports.^2^

Di Biase et al reported that RFCA may be unsuccessful at the site of the earliest local ventricular activation when the bipolar electrogram is far field. Activation mapping (using the maximal negative *dV/dt* of the unipolar electrogram and the maximal amplitude of the bipolar electrogram) must be performed to define the real origin of the VA.^3^ In our patient, PVCs could not be eliminated in the RVOT, as the earliest bipolar electrogram appeared to exhibit low‐amplitude and slow‐frequency far‐field potential. Ablation had not been applied near the ostium of the left main coronary artery, where the earliest far‐field bipolar electrogram was mapped and the RF energy possibly resulted in a lesion to artery. At the bottom of left sinus of Valsalva, the local ventricular activation preceding the QRS onset of the PVCs was not the earliest, but it showed the breakout site of PVCs, which should be of intramural origin. Successful RFCA was achieved by deciding to systematically apply sequential, unipolar ablation at the RVOT, the left sinus of Valsalva, and the subaortic valvular region.^3^

Bloch Thomsen et al^4^ reported that the LVP of the ventricular output tract may reflect an area of depressed conductivity known to be a prerequisite for ventricular ectopy. In our patient, on the basis of potential characteristics, electrical impulses of PVC origin had conducted slowly into the breakout site in the bottom of the left sinus of Valsalva, and there was an isoelectric line between the LVP and the ventricular potential. We speculate that the LVP represents the area of impaired and anisotropic conduction. This suggests that conduction velocities in the ventricular outflow tract muscle are not fixed, but rather dependent of wavefront propagation. The variation in conduction velocities accounts for preferential conduction to multiple exit sites.

In short, this case suggests that distinguishing near‐field from far‐field potentials is helpful for identifying the real site of origin of ventricular arrhythmia. The phenomenon of preferential conduction to multiple exit sites may occur in the LVOT when conduction is impaired and during anisotropic conduction of the muscle surrounding the site of origin.

## Supporting information

Video 1. Fluoroscopic documentation: Radiofreqency catheter was close to the ostium of the left main coronary artery

Video 2. Video from carto system: The PVCs with the second QRS morphology was eliminated, the spontaneous PVCs changed to the third form
